# The levels of women’s awareness, experience, acceptability and preference for Vaginal Human Papillomavirus (HPV) self-sampling in three provinces of China: a cross-sectional study

**DOI:** 10.1186/s12905-024-03186-w

**Published:** 2024-06-15

**Authors:** Jia Song, Yi-Hua Ni, Jing Fang, Shui-Xiang Qu, Xiao-Yan Chen, Wei-Li Wu, Wei-Chu Zhang, Jian-Fen Qin

**Affiliations:** Nursing Department, Sir Run Run Shaw Hospital, Zhejiang University School of Medicine, Hangzhou, 310020 China

**Keywords:** Human papillomavirus, Self-sampling, Awareness, Experience, Acceptability, Preference

## Abstract

**Background:**

The primary screening technique for precancerous lesions and cervical cancer is human papillomavirus (HPV) testing, and HPV self-sampling has been shown to be consistent with clinician sampling in terms of the accuracy of the results and may improve cervical cancer screening rates. The aim of this study was to understand the level of awareness, experience, acceptability, and preference for vaginal HPV self-sampling among women in Jiangsu, Zhejiang, and Shanghai, China, and to analyze the possible influencing factors to determine the feasibility of implementing self-sampling.

**Methods:**

Overall, 1793 women were included in the data analysis. A self-administered questionnaire was utilized. In addition to descriptive analysis, univariate and multivariate analyses were used to explore the associations between sociodemographic features, history of cervical cancer screening, and the level of awareness, experience, acceptability, and preference for HPV self-samples.

**Results:**

The participants’ level of awareness of and experience with HPV self-sampling were moderate. A total of 88.8% of participants rated the acceptability as “high”, and self-sampling was preferred by 64.2% of them for cervical cancer screening. People aged 45 to 54 years showed a preference for both clinician sampling(*OR* = 1.762 (1.116–2.163)) and self-sampling (*OR* = 1.823 (1.233–2.697)). Those who had graduated from high school or above (*OR* = 2.305 (1.517–3.503), *OR* = 2.432 (1.570–3.768), *OR* = 3.258 (2.024–5.244)) preferred clinician-sampling, and those with a bachelor’s degree or above (*OR* = 1.664 (1.042–2.657)) preferred self-sampling. Middle- and high-income individuals showed no preference for either sampling method (*OR* < 1).

**Conclusions:**

HPV self-sampling is widely accepted, but awareness, experience and preferences need to be improved. These results may help to adjust public health strategies for the early inclusion of HPV self-sampling as a screening method in national initiatives to prevent cervical cancer.

## Background

As a typical malignancy in women, cervical cancer has a high incidence and mortality rate. According to the data provided by the International Agency for Research on Cancer (IARC) of the World Health Organization (WHO), in 2020, there were 604,000 new cases of cervical cancer worldwide and 342,000 deaths from the disease; these are the 4th highest incidence rate and 4th highest cause of death from common female malignant tumors in the world [1]. Over the past two to three decades, the incidence and mortality of cervical cancer in developed nations have declined significantly, which is mainly attributed to the application of prophylactic HPV vaccinations and organized screening for cervical cancer [2].

Cervical cancer screening can swiftly prevent aggressive cervical cancer and precancerous changes in women, and the WHO promotes cervical cancer screening globally for detection and treatment at an early stage. In 2021, the WHO published guidelines for the screening and treatment of cervical precancerous lesions, which explicitly recommend tests for HPV DNA as the first line of screening, suggesting that samples collected by healthcare providers or self-collected by women be used for testing [3]. It is also noted that self-collected samples have a high level of efficacy and acceptance, which reduces cultural and socioeconomic barriers to screening and increases equity. Currently, structured cervical screening programs in many nations such as Norway, Denmark, and the United Kingdom involve self-sampling [4]. Several studies have indicated that when polymerase chain reaction (PCR) is used for HPV DNA testing, cervical samples taken by the patients themselves and samples collected by a physician yield consistent findings [5, 6], which provides a sound scientific basis for promoting HPV self-sampling. In addition, HPV self-sampling has the benefits of simplicity of usage, privacy, convenience, bodily and mental comfort (including less pain, worry, and shame), and time savings for medical appointments [7], which may increase the motivation of women who are underscreened to engage in screening, consequently increasing screening uptake. However, implementing HPV self-sampling also requires overcoming a number of barriers, including women’s mistrust of their capacity to appropriately collect samples and the accuracy of the outcomes, as well as fear of injuring themselves by mistake [8].

As a developing country, China has not yet established a full-coverage cervical cancer screening system at this stage, and the cervical cancer screening coverage percentage is relatively low. Studies have shown that the screening rate of women aged 35–49 years in China in the past five years has been 33% [9], which is much lower than the 70% screening rate in the WHO target [10]. In terms of cervical cancer screening methods, China has reacted vigorously to the relevant guidelines, and the latest expert consensus developed in China clearly recommends HPV nucleic acid testing as the main technique for screening for cervical cancer and highlights that exploring the implementation strategy of applying HPV self-sampling methods to screening practice is essential [11]. Adequate knowledge and positive attitudes are the basis of successful practices [12], and it is crucial to understand the knowledge and attitudes of people from different cultural backgrounds toward HPV self-sampling. In addition, several studies have shown that sociodemographic characteristics such as age, ethnicity, religion, education, economic income, and the duration of cervical cancer screening greatly influence the feasibility and acceptability of HPV self-sampling [13–15]. Jiangsu, Zhejiang, and Shanghai, which are more developed regions in China in terms of economic, technological, and medical development, provide favorable external conditions for HPV self-sampling. The specific associations of factors such as age, schooling, occupation, marriage and childbearing background and history of cervical cancer tests with women’s knowledge and attitudes about HPV self-sampling in Jiangsu, Zhejiang, and Shanghai, China, are not yet known. To explore potential differences between our findings and those of previous domestic and international studies and to determine whether self-sampling of HPV is feasible, we surveyed women who had completed vaginal HPV self-sampling in Jiangsu, Zhejiang, and Shanghai, China, to understand their level of awareness, experience, acceptability, and preference for self-sampling and to analyze the factors influencing them.

## Methods

### Settings

This was an anonymous cross-sectional study conducted from July 2022 to November 2022 in Zhejiang, Jiangsu, and Shanghai, China.

### Participants and testing methods

Vaginal HPV self-sampling is an approach in which the subject collects exfoliated cells from the vaginal cervix by themselves. Our research group has developed an integrated HPV self-sampling testing platform, which has three ports—the user side, the testing side, and the healthcare side—and includes the functions of user access to self-sampling tools, sample logistics tracking, testing services, report queries, medical consulting, healthcare management, and follow-up tracking, etc [16]. In this study, we used the “microcollection HPV” special self-sampling brush (developed by Shanghai Zhijiang Biotechnology Co., Ltd, registration certificate No. Zhexiezhuzhun:20,162,411,052) for HPV sampling, and applied fluorescence quantitative PCR detection [a high-risk human papillomavirus (HPV) typing nucleic acid assay reagent kit (No. Guoxiezhuzhun: 20,153,400,044)], with a Chinese version of the self-sampling procedure guidance material attached to the kit. A convenience sampling method was used to select women who purchased HPV self-sampling kits and completed HPV testing by information registered on this platform. Because the best age to start screening for cervical cancer is unclear and recommendations vary among expert panels, this study synthesized relevant guidelines to include women aged 21 and older [3, 17–19].Women who had not had sexual intercourse, were pregnant, had been diagnosed with cervical cancer, and had received treatment for cervical abnormalities; who were illiterate, had mental retardation, or had other conditions that affect cognitive functioning; or who had poor compliance and inability to fill out the questionnaire completely were excluded.

### Questionnaire

Through a literature review and expert correspondence [20, 21], this study designed its own questionnaire on the level of awareness, experience, acceptability and preference for self-sampling of HPV, which consisted of four parts with a total of 42 entries. The first part (Questions 1 to 15) included sociodemographic information (including age, height, weight, education level, marriage and childbearing history, abortion history, per capita monthly household income, occupation, etc.) and history of cervical cancer screening. The second section (Questions 16 to 22) addressed the level of awareness about cervical cancer screening and HPV self-sampling. A five-point Likert scale was used, with each question having a score between one and five and a total score ranging from 7 to 35; the overall score was used to categorize knowledge into three levels: low level of awareness (≤ 21 points), moderate level of awareness (22 to 28 points), and high level of awareness (29 to 35 points). The third section (Questions 23 to 31) utilized a five-point Likert scale to assess the self-sampling experience of HPV. The final two questions used inversion scoring, with a total score ranging from 9 to 45 points. According to the total score of the experience degree, the experience degree was divided into three grades: poor experience (≤ 27 points), moderate experience (28 to 36 points) and good experience (37 to 45 points). The fourth part (Questions 32 to 41) concerned HPV self-sampling acceptability and preference, and Question 32 concerned the degree of acceptability; these questions were categorized by a cutoff of 3 points into grades of low acceptability (1 to 2 points), medium acceptability (3 points), and high acceptability (4 to 5 points). The last question (Question 42) was an open-ended question for participants asking questions or making suggestions.

The psychometric properties of this questionnaire were verified by its internal consistency and stability of the questionnaire. In this study, 420 women who met the inclusion criteria were preselected for presurvey, and 410 valid questionnaires were ultimately included for analysis. The Cronbach’s alpha reliability coefficient (Cronbach’s α) was used to measure the consistency among the questionnaire items, and a general reliability coefficient of 0.7 or above indicates a high degree of internal consistency of the questionnaire [22]. The total Cronbach’s alpha coefficient of the three parts of this questionnaire, awareness level, experience, acceptability and preference for HPV self-sampling, is 0.924, which indicates a high degree of internal consistency. Retest reliability is often used to indicate the stability of a measurement instrument, i.e., the degree of consistency of the results obtained by using the same instrument to measure the same group of research subjects two or more times. The typical retesting time was 2 weeks after the presurvey. The higher the retest reliability is, the better the stability of the research instrument [23]. In this study, 100 women who received the presurvey were selected for the survey to be surveyed using the same questionnaire after a 2-week interval, and the retest reliability of this questionnaire was analyzed to be 0.956, which indicates that the retest reliability is high. Therefore, this questionnaire has good internal consistency and stability.

### Data collection

In this study, an electronic questionnaire was used to generate a link to the questionnaire through an online survey platform called “Wenjuanxing”. Women who satisfied the requirements for inclusion were selected from the self-sampling platform’s backend, and a link to the questionnaire was sent to them by text message with an explanation of the purpose of the questionnaire and the content of the study. If participants had any questions, they could contact the researcher by e-mail or phone. Every participant in the study was free to choose whether to participate, and they could leave at any time. A page outlining the goal of the study was included, and participants’ informed consent was requested before the questionnaire could be returned. Participants could finish the questionnaire if they provided informed consent. Each question in the electronic version of the questionnaire was set as mandatory, and each questionnaire was checked by the researcher for quality of completion, excluding logical errors and questionnaires that took too little time to complete (less than 3 min). To ensure the response rate of the questionnaire, the QR code of the questionnaire was synchronously attached to the self-sampling instructions in the kit; a link to the questionnaire was attached to the test report when it was sent; and those who did not complete the questionnaire in time were followed up by telephone and reminded to complete the questionnaire again. A total of 2,020 questionnaires were eventually distributed, and 1,796 completed questionnaires were returned, for a response rate of 88.9%.

### Data analysis

The data were entered and organized using Excel, and the statistical analysis was performed using SPSS 26.0. The mean and standard deviation were used to represent normally distributed measurement information; the median and quartile were used for describing data that were not normally distributed; and the number of cases and percent were used to express counting information. The HPV self-sampling awareness and experience scores were verified not to conform to a normal distribution, while the acceptability score was based on the hierarchical information; therefore, the Kruskal—Wallis H test of nonparametric tests was applied to the level of awareness, experience, and acceptability; the *x*^*2*^ test was applied to the preference; and univariate analysis was carried out. Subsequently, the multivariate analysis included variables from the univariate analysis that were of statistical significance, and multivariate linear regression was used for continuous numerical variables (the level of awareness and experience); multivariate logistic regression was used for subtyped variables (acceptability and preference). *P* < 0.05 was considered to indicate statistical significance.

## Results

### Sociodemographic features and current status of preference

This study included a total of 1793 valid questionnaires, and 3 questionnaires with invalid responses were excluded (1 took less than 3 min to complete, and 2 had logical errors). Among the participants, 1,193 (66.5%) were from Jiangsu, 517 (28.9%) were from Zhejiang, and 83 (4.6%) were from Shanghai. Ages ranged from 21 to 65 years, with an average age of 41.25 ± 7.793 years. The mean BMI was 22.53 ± 2.87, with 69.6% (1,248) having a normal body mass index (BMI). A total of 51.2% (918) had an associate or bachelor’s degree or above. Married (93.5%, 1676) and childbearing (96.3%, 1726) women were the predominant participants. The majority (78.4%, 1406) had a monthly income per household of 1,000–5,000 RMB. The occupations of the participants were mainly workers (40.7%, 729) and company employees (32.9%, 590). A total of 63.4% (1,137) had undergone screening for cervical cancer within the previous year, while 11.5% (207) had not undergone cervical cancer screening. Of the 1,586 participants who had been screened for cervical cancer, the vast majority (78.8%, 1,249) had an HPV test, and 89.0% (1,412) had a normal screening result. Of the 65 participants with abnormal cervical cancer screening results, 87.7% (57) had a positive HPV test result. In terms of preference, of the similar number of participants, 641 (35.8%) and 644 (35.9%) chose clinician sampling and self-sampling, respectively, while the other 508 (28.3%) had no preference. The majority of participants (76.9%, 1378) were ready to introduce others to HPV self-sampling. Table 1 displays the abovementioned sociodemographic features and preferences.

**Table 1:** Sociodemographic features and preferences for HPV self-sampling

Variables	*N* (%) or Mean (SD)	Variables	*N* (%)
**Age(years)**	*N* = 1793	**How much did you spend on a cervical cancer screening?**	*N* = 1586
Mean (SD)	41.25(7.793)	For free	721(45.5%)
≤ 34	431(24.0%)	<100RMB	13(0.8%)
35–44	664(37.0%)	100-200RMB	67(4.2%)
45–54	627(35.0%)	>200RMB	328(20.7%)
≥ 55	71(4.0%)	Unclear	457(28.8%)
**BMI** ^**1**^	*N* = 1793	**How long did you spend on a cervical cancer screening?**	*N* = 1586
Mean (SD)	22.53(2.87)	<1 h	842(53.1%)
<18.5	80(4.5%)	1–2 h	321(20.2%)
18.5–23.9	1248(69.6%)	3–4 h	125(7.9%)
24-27.9	373(20.8%)	>4 h	298(18.8%)
≥ 28	92(5.1%)	**What is your preferred time frame for a report on HPV self-sampling testing?**	*N* = 1793
**Education**	*N* = 1793	Within 4 days	797(44.5%)
Primary school or below	85(4.7%)	5–7 days	862(48.1%)
Junior High School	426(23.8%)	8–10 days	85(4.7%)
High school or vocational school	364(20.3%)	11–15 days	49(2.7%)
Associate degree	464(25.9%)	**Which method do you think is more accurate in terms of test results, self-sampling or clinician-sampling?**	*N* = 1793
Bachelor degree or above	454(25.3%)	Clinician-sampling	670(37.4%)
**Marital status**	*N* = 1793	Self-sampling	161(9.0%)
Single	36(2.0%)	They’re both equally accurate.	698(38.9%)
Married	1676(93.5%)	Unclear	264(14.7%)
Divorced	65(3.6%)	**Which kind of usual cervical cancer screening do you prefer?**	*N* = 1793
Widowed	16(0.9%)	Clinician-sampling	641(35.8%)
**Fertility status**	*N* = 1793	Self-sampling	644(35.9%)
No child	67(3.7%)	No preference	508(28.3%)
Have a child	1088(60.7%)	**Reasons to select clinician-sampling**	*N* = 1149
Have 2 children or above	638(35.6%)	More accurate sampling	831(72.3%)
**Abortion history**	*N* = 1793	Clinicians can see more information about diseases	823(71.6%)
Never	665(37.1%)	More reliable results	510(44.4%)
1 abortion	674(37.6%)	Traditional perceptions	143(12.4%)
2 abortions or above	454(25.3%)	Others	9(0.8%)
**Monthly income per household**	*N* = 1793	**Reasons to select self-sampling**	*N* = 1152
<1000 RMB	46(2.6%)	Convenient and easy to operate	884(76.7%)
1000–3000 RMB	726(40.5%)	Better protection of privacy	814(70.7%)
3001–5000 RMB	680(37.9%)	Reduction of pain and fear of gynecological speculum	598(51.9%)
>5000 RMB	341(19.0%)	Freedom of time and place	136(11.8%)
**Occupations**	*N* = 1793	Others	13(1.1%)
Professional	184(10.3%)	**Would you like to introduce others the HPV self-sampling test?**	*N* = 1793
Service industry personnel	73(4.1%)	Very unwilling	49(2.7%)
Worker	729(40.7%)	Unwilling	64(3.6%)
Company employee	590(32.9%)	Just so so	302(16.8%)
Public functionary	71(4.0%)	Willing	672(37.5%)
Housewife	28(1.5%)	Very willing	706(39.4%)
Retire	46(2.5%)	**What are you prepared to pay for an HPV self-sampling test?**	*N* = 1793
Others	72(4.0%)	<100 RMB	1214(67.7%)
**Have you participated in the cervical cancer screening program conducted by the government or community?**	*N* = 1793	100–200 RMB	432(24.1%)
Unheard	377(21.0%)	201–300 RMB	90(5.0%)
Heard of it, not participated	582(32.5%)	>300 RMB	57(3.2%)
Participated	834(46.5%)	**Where would you prefer to have an HPV self-sampling test?**	*N* = 1793
**How recently did you get screened for cervical cancer?**	*N* = 1793	Hospital	708(39.5%)
<1 year	1137(63.4%)	Home	1011(56.4%)
1–2 years	330(18.4%)	Others	74(4.1%)
3–5 years	60(3.3%)	**What aspect of the HPV self-sampling test worries you most?**	*N* = 1793
>5 years	20(1.2%)	Deteriorated or contaminated specimens	1007(56.2%)
Never done it before	207(11.5%)	Incorrect sampling	931(51.9%)
Unclear	39(2.2%)	Inaccurate results	529(29.5%)
**What was the outcome of the most recent cervical cancer screening?**	*N* = 1586	Results not available in time	430(24.0%)
Negative	1412(89.0%)	Lack of interpretation of the results and advice on diagnosis and treatment	290(16.2%)
Positive	82(5.2%)	Injuring themselves by mistake during sampling	270(15.1%)
Unclear	92(5.8%)	Others	47(2.6%)

### Current status of the level of awareness, experience, and acceptability of HPV self-sampling

In this study, the mean overall awareness score for HPV self-sampling was 24.6 (95% CI 24.3–24.9), and the average number of experience points was 35.4 (95% CI 35.1–35.6). The mean acceptability point was 4.20 (95% CI 4.16–4.24), and 88.8% (1413) of the participants rated self-sampling acceptability as “high”. We found that the participants had moderate levels of awareness and experience and high levels of acceptability. In terms of awareness, the question “Do you know about self-sampling practices for HPV?” had the highest score of 3.69 (95% CI 3.64–3.74), and the question “Do you know the process for further management of HPV positivity?” had the lowest score of 3.11 (95% CI 3.06–3.17). In terms of experience, “level of privacy” had the highest rating (4.30; 95% CI 4.26–4.34), and “level of pain” had the lowest rating (3.03; 95% CI 2.96–3.10). Table 2 displays the participants’ awareness, experience, and acceptability of HPV self-sampling.

**Table 2:** The level of awareness, experience, and acceptability of HPV self-sampling

Variables	Mean (95% CI)^a^	Median (*P*_25,_ *P*_75_)^b^
**Awareness about HPV self-sampling and cervical cancer**		
Do you understand the dangers of cervical cancer?	3.66(3.62,3.71)	4(3,4)
Do you understand the meaning of the HPV testing?	3.63(3.58,3.68)	4(3,4)
Do you understand the dangers of being HPV positive?	3.58(3.53,3.63)	4(3,4)
Do you know the process for further management of HPV positivity?	3.11(3.06,3.17)	3(2,4)
Do you know the HPV self-sampling test?	3.56(3.50,3.61)	4(3,4)
Do you know the HPV Network Self-sampling Test?	3.36(3.30,3.41)	3(2,4)
Do you know about self-sampling practices for HPV?	3.69(3.64,3.74)	4(3,5)
Total scores	24.6(24.3,24.9)	25(21,28)
**Experience of HPV self-sampling**		
Overall satisfaction with HPV self-sampling	4.03(3.99,4.07)	4(4,5)
Satisfaction with the user registration process of the HPV self-sampling test platform	4.11(4.07,4.14)	4(4,5)
Satisfaction with the HPV self-sampling operation video and instructions	4.17(4.14,4.21)	4(4,5)
Was your sampling procedure (the process of completing the sample with the sampling tool) smooth?	4.22(4.18,4.25)	4(4,5)
Satisfaction with the way you access the report for the HPV self-sampling test	4.17(4.14,4.21)	4(4,5)
Rate your convenience for the HPV self-sampling test	4.28(4.24,4.32)	4(4,5)
Rate your level of privacy for the HPV self-sampling test	4.30(4.26,4.34)	5(4,5)
Rate your level of pain for the HPV self-sampling test	3.03(2.96,3.10)	3(2,4)
Rate your embarrassment about the HPV self-sampling test	3.08(3.01,3.15)	3(2,4)
Total scores	35.4(35.1,35.6)	36(32,39)
**Acceptability of HPV self-sampling**		
The HPV self-sampling acceptability score	4.20(4.16,4.24)	4(4,5)

^a^95% CI: 95% confidence interval

^b^P_25_: the first quartile, P_75_: the third quartile

### Results of univariate analysis of HPV self-sampling awareness, experience, acceptability and preference

HPV self-sampling awareness and experience score were used as dependent variables, and the sociodemographic features of the research subjects were used as the independent variables for univariate analysis. Acceptability and preference were used as dependent variables, and participants’ sociodemographic features and HPV self-sampling awareness and experience score were used as the independent variables for univariate analysis. The results are shown in Tables 3 and 4.

**Table 3:** Univariate analysis of the level of awareness and experience of HPV self-sampling

Variables		Awareness			Experience		
		Score *M*(*P*_25_,*P*_75_)	*H*	*P value*	Score *M*(*P*_25_,*P*_75_)	*H*	*P value*
Age	≤ 34	26.00(21.00,29.00)	6.333	0.096	36.00(32.00,39.00)	0.731	0.866
	35–44	24.00(21.00,28.00)			36.00(32.00,39.00)		
	45–54	24.00(20.00,28.50)			36.00(32.00,39.00)		
	≥ 55	22.00(17.00,35.00)			37.00(32.00,37.00)		
BMI	<18.5	26.50(21.00,29.00)	2.961	0.398	37.00(32.00,37.00)	0.017	0.999
	18.5–23.9	24.00(21.00,28.00)			36.00(32.00,39.00)		
	24-27.9	25.00(20.00,29.00)			36.00(32.00,39.00)		
	≥ 28	24.00(20.50,28.00)			36.00(32.00,39.00)		
Education	Primary school or below	21.00(14.00,28.00)	95.640	**<0.001**	36.00(31.00,37.00)	19.590	**0.001**
	Junior High School	21.00(17.00,28.00)			35.00(31.00,37.00)		
	High school or vocational school	25.00(21.00,28.00)			36.00(32.00,41.00)		
	Associate degree	26.00(21.00,29.00)			36.00(32.00,39.00)		
	Bachelor degree or above	27.00(22.00,30.00)			36.00(32.00,40.00)		
Marital status	Single	26.50(21.00,30.00)	5.738	0.125	32.50(27.00,37.00)	11.387	**0.010**
	Married	25.00(21.00,28.00)			36.00(32.00,39.00)		
	Divorced	26.00(21.00,33.00)			37.00(32.00,40.00)		
	Widowed	21.00(18.50,29.50)			32.50(29.50,37.00)		
Fertility status	No child	26.00(21.00,28.50)	9.948	**0.007**	33.00(28.00,37.00)	14.902	**0.001**
	Have a child	25.00(21.00,29.00)			36.50(32.00,39.00)		
	Have 2 children or above	24.00(21.00,28.00)			35.00(32.00,39.00)		
Abortion history	Never	26.00(21.00,29.00)	8.256	**0.016**	36.00(32.00,38.00)	0.711	0.701
	1 abortion	24.00(20.00,28.00)			36.00(32.00,39.00)		
	2 abortions or above	24.00(20.00,29.00)			36.00(32.00,40.00)		
Monthly income per household	<1000RMB	21.00(14.00,30.00)	9.889	**0.020**	32.00(27.00,37.00)	12.366	**0.006**
	1000-3000RMB	24.00(21.00,28.00)			36.00(32.00,39.00)		
	3001-5000RMB	25.00(21.00,29.00)			36.00(32.00,39.00)		
	>5000RMB	25.00(21.00,29.00)			37.00(32.00,40.00)		
Occupations	Professional	28.00(22.00,32.00)	57.522	**<0.001**	36.00(32.00,40.00)	4.139	0.764
	Service industry personnel	25.00(21.00,28.00)			36.00(32.00,38.00)		
	Worker	24.00(20.00,28.00)			35.00(32.00,39.00)		
	Company employee	24.00(21.00,28.00)			36.00(32.00,39.00)		
	Public functionary	26.00(22.50,32.50)			36.00(32.00,38.50)		
	Housewife	22.00(15.00,29.00)			36.50(33.50,37.00)		
	Retire	33.50(21.00,35.00)			37.00(34.00,37.00)		
	Others	24.50(21.00,30.50)			37.00(32.00,40.00)		
Have you participated in the cervical cancer screening program conducted by the government or community?	Unheard	21.00(17.00,27.00)	123.532	**<0.001**	34.00(31.00,38.00)	16.329	**<0.001**
	Heard of it, not participated	24.00(20.00,28.00)			36.00(32.00,39.00)		
	Participated	27.00(22.00,31.00)			37.00(32.00,39.00)		
How recently did you get screened for cervical cancer?	<1 year	26.00(21.00,30.00)	177.849	**<0.001**	36.00(32.00,39.00)	37.734	**<0.001**
	1–2 years	24.00(21.00,29.00)			36.00(32.00,39.00)		
	3–5 years	21.00(18.00,27.00)			35.50(32.00,38.00)		
	>5 years	21.00(14.50,25.50)			33.00(30.00,37.00)		
	Never done it before	20.00(15.00,24.00)			34.00(29.00,38.00)		
	Unclear	17.00(14.00,23.50)			32.00(28.50,36.50)		
What was the outcome of the most recent cervical cancer screening? (*n* = 1586)	Negative	26.00(21.00,29.00)	154.822	**<0.001**	36.00(32.00,39.00)	34.335	**<0.001**
	Positive	25.00(21.00,29.00)			36.00(32.00,39.00)		
	Unclear	19.00(14.00,25.50)			33.00(27.00,37.50)		
How much did you spend on a cervical cancer screening? (*n* = 1586)	For free	27.00(21.00,31.00)	163.636	**<0.001**	37.00(32.00,40.00)	43.575	**<0.001**
	<100RMB	21.00(15.00,27.00)			32.00(31.00,34.00)		
	100-200RMB	23.00(21.00,28.00)			33.00(31.00,37.00)		
	>200RMB	26.00(21.00,29.00)			35.00(32.00,39.00)		
	Unclear	23.00(19.00,28.00)			35.00(32.00,38.00)		
How long did you spend on a cervical cancer screening? (*n* = 1586)	<1 h	26.00(21.00,30.00)	139.365	**<0.001**	36.00(32.00,39.00)	19.771	**0.001**
	1–2 h	26.00(21.00,29.00)			36.00(32.00,39.00)		
	3–4 h	21.00(18.00,27.00)			34.00(32.00,39.00)		
	>4 h	25.00(21.00,28.00)			36.00(32.00,39.00)		

**Table 4:** Univariate analysis of HPV self-sampling acceptability and preferences

Variables	Score M(*P*_25_,*P*_75_)	H	*P* value ^a^	Clinician-sampling *n*(c)/(d)	Self-sampling *n*(c)/(d)	No preference *n*(c)/(d)	x ^2^	*P* value ^b^
Age								
≤ 34	4.00(4.00,5.00)	1.170	0.760	139(21.7%)/ (32.3%)	171(26.6%)/ (39.7%)	121(23.8%)/ (28.1%)	34.675	**<0.001**
35–44	4.00(4.00,5.00)			248(38.7%)/ (37.3%)	242(37.6%)/ (36.4%)	174(34.3%)/ (26.2%)		
45–54	4.00(4.00,5.00)			230(35.9%)/ (36.7%)	190(29.5%)/ (30.3%)	207(40.7%)/ (33.0%)		
≥ 55	5.00(4.00,5.00)			24(3.7%)/ (33.8%)	41(6.4%)/ (57.7%)	6(1.2%)/ (8.5%)		
BMI								
<18.5	5.00(4.00,5.00)	4.278	0.233	27(4.2%)/ (33.8%)	32(5.0%)/ (40.0%)	21(4.1%)/ (26.3%)	11.044	0.087
18.5–23.9	4.00(4.00,5.00)			450(70.2%)/ (36.1%)	464(72.0%)/(37.2%)	334(65.7%)/(26.8%)		
24-27.9	4.00(4.00,5.00)			138(21.5%)/ (37.0%)	118(18.3%)/ (31.6%)	117(23.0%)/ (31.4%)		
≥ 28	4.00(4.00,5.00)			26(4.1%)/ (28.3%)	30(4.7%)/ (32.6%)	36(7.1%)/ (39.1%)		
Education								
Primary school or below	5.00(4.00,5.00)	4.773	0.311	41(6.4%)/ (48.2%)	43(6.7%)/ (50.6%)	1(0.2%)/ (1.2%)	88.903	**<0.001**
Junior High School	4.00(4.00,5.00)			210(32.8%)/ (49.3%)	131(20.3%)/ (30.8%)	85(16.7%)/ (20.0%)		
High school or vocational school	4.00(4.00,5.00)			113(17.6%)/ (31.0%)	129(20.0%)/ (35.4%)	122(24.0%)/ (33.5%)		
Associate degree	4.00(4.00,5.00)			135(21.1%)/ (29.1%)	183(28.4%)/ (39.4%)	146(28.7%)/ (31.5%)		
Bachelor degree or above	4.00(4.00,5.00)			142(22.2%)/ (31.3%)	158(24.5%)/ (34.8%)	154(30.3%)/ (33.9%)		
Marital status								
Single	4.00(3.00,5.00)	8.361	**0.039**	10(1.6%)/ (27.8%)	13(2.0%)/ (36.1%)	13(2.6%)/ (36.1%)	4.893	0.558
Married	4.00(4.00,5.00)			605(94.4%)/ (36.1%)	603(93.6%)/ (36.0%)	468(92.1%)/ (27.9%)		
Divorced	5.00(4.00,5.00)			19(3.0%)/ (29.2%)	25(3.9%)/ (38.5%)	21(4.1%)/ (32.3%)		
Widowed	4.00(3.00,5.00)			7(1.1%)/ (43.8%)	3(0.5%)/ (18.8%)	6(1.2%)/ (37.5%)		
Fertility status								
No child	4.00(3.00,5.00)	12.212	**0.002**	21(3.3%)/ (31.3%)	23(3.6%)/ (34.3%)	23(4.5%)/ (34.3%)	10.770	**0.029**
Have a child	4.00(4.00,5.00)			366(57.1%)/ (33.6%)	392(60.9%)/ (36.0%)	330(65.0%)/ (30.3%)		
Have 2 children or above	4.00(4.00,5.00)			254(39.6%)/ (39.8%)	229(35.6%)/ (35.9%)	155(30.5%)/ (24.3%)		
Abortion history								
Never	4.00(4.00,5.00)	1.769	0.413	197(30.7%)/ (29.6%)	262(40.7%)/ (39.4%)	206(40.6%)/ (31.0%)	18.616	**0.001**
1 abortion	4.00(4.00,5.00)			268(41.8%)/ (39.8%)	234(36.3%)/ (34.7%)	172(33.9%)/ (25.5%)		
2 abortions or above	4.00(4.00,5.00)			176(27.5%)/ (38.8%)	148(23.0%)/ (32.6%)	130(25.6%)/ (28.6%)		
Monthly income per household								
<1000RMB	4.00(3.00,5.00)	1.485	0.686	25(3.9%)/ (54.3%)	14(2.2%)/ (30.4%)	7(1.4%)/ (15.2%)	40.804	**<0.001**
1000–3000 RMB	4.00(4.00,5.00)			236(36.8%)/ (32.5%)	240(37.3%)/ (33.1%)	250(49.2%)/ (34.4%)		
3001–5000 RMB	4.00(4.00,5.00)			267(41.7%)/ (39.3%)	234(36.3%)/ (34.4%)	179(35.2%)/ (26.3%)		
>5000 RMB	4.00(4.00,5.00)			113(17.6%)/ (33.1%)	156(24.2%)/ (45.7%)	72(14.2%)/ (21.1%)		
Occupations								
Professional	4.00(4.00,5.00)	13.695	0.057	77(12.0%)/ (41.8%)	50(7.8%)/ (27.2%)	57(11.2%)/ (31.0%)	69.414	**<0.001**
Service industry personnel	4.00(3.00,5.00)			26(4.1%)/ (35.6%)	17(2.6%)/ (23.3%)	30(5.9%)/ (41.1%)		
Worker	4.00(4.00,5.00)			237(37.0%)/ (32.5%)	253(39.3%)/ (34.7%)	239(47.0%)/ (32.8%)		
Company employee	4.00(4.00,5.00)			234(36.5%)/ (39.7%)	225(34.9%)/ (38.1%)	131(25.8%)/ (22.2%)		
Public functionary	5.00(4.00,5.00)			17(2.7%)/ (23.9%)	24(3.7%)/ (33.8%)	30(5.9%)/ (42.3%)		
Housewife	5.00(4.00,5.00)			12(1.9%)/ (42.9%)	14(2.2%)/ (50.0%)	2(0.4%)/ (7.1%)		
Retire	5.00(4.00,5.00)			11(1.7%)/ (23.9%)	31(4.8%)/ (67.4%)	4(0.8%)/ (8.7%)		
Others	5.00(4.00,5.00)			27(4.2%)/ (37.5%)	30(4.7%)/ (41.7%)	15(3.0%)/ (20.8%)		
Have you participated in the cervical cancer screening program conducted by the government or community?								
Unheard	4.00(4.00,5.00)	13.115	**0.001**	160(25.0%)/ (42.4%)	141(21.9%)/ (37.4%)	76(15.0%)/ (20.2%)	24.450	**<0.001**
Heard of it, not participated	4.00(4.00,5.00)			221(34.5%)/ (38.0%)	195(30.3%)/ (33.5%)	166(32.7%)/ (28.5%)		
Participated	5.00(4.00,5.00)			260(40.6%)/ (31.2%)	308(47.8%)/ (36.9%)	266(52.4%)/ (31.9%)		
How recently did you get screened for cervical cancer?								
<1 year	4.00(4.00,5.00)	29.067	**<0.001**	345(53.8%)/ (30.3%)	398(61.8%)/ (35.0%)	394(77.6%)/ (34.7%)	81.633	**<0.001**
1–2 years	4.00(4.00,5.00)			132(20.6%)/ (40.0%)	125(19.4%)/ (37.9%)	73(14.4%)/ (22.1%)		
3–5 years	4.00(4.00,5.00)			28(4.4%)/ (46.7%)	23(3.6%)/ (38.3%)	9(1.8%)/ (15.0%)		
>5 years	4.00(4.00,5.00)			11(1.7%)/ (55.0%)	7(1.1%)/ (35.0%)	2(0.4%)/ (10.0%)		
Never done it before	4.00(3.00,5.00)			103(16.1%)/ (49.8%)	79(12.3%)/ (38.2%)	25(4.9%)/ (12.1%)		
Unclear	4.00(3.00,5.00)			22(3.4%)/ (56.4%)	12(1.9%)/ (30.8%)	5(1.0%)/ (12.8%)		
What was the outcome of the most recent cervical cancer screening? (*n* = 1586)								
Negative	4.00(4.00,5.00)	32.820	**<0.001**	460(85.5%)/ (32.6%)	515(91.2%)/ (36.5%)	437(90.5%)/ (30.9%)	47.569	**<0.001**
Positive	4.00(4.00,5.00)			36(6.7%)/ (43.9%)	20(3.5%)/ (24.4%)	26(5.4%)/ (31.7%)		
Unclear	4.00(3.00,5.00)			42(7.8%)/ (45.7%)	30(5.3%)/ (32.6%)	20(4.1%)/ (21.7%)		
How much did you spend on a cervical cancer screening? (*n* = 1586)								
For free	5.00(4.00,5.00)	34.327	**<0.001**	199(37.0%)/ (27.6%)	270(47.8%)/ (37.4%)	252(52.2%)/ (35.0%)	110.200	**<0.001**
<100RMB	4.00(4.00,5.00)			7(1.3%)/ (53.8%)	5(0.9%)/ (38.5%)	1(0.2%)/ (7.7%)		
100-200RMB	4.00(4.00,5.00)			42(7.8%)/ (62.7%)	21(3.7%)/ (31.3%)	4(0.8%)/ (6.0%)		
>200RMB	4.00(4.00,5.00)			144(26.8%)/ (43.9%)	119(21.1%)/ (36.3%)	65(13.5%)/ (19.8%)		
Unclear	4.00(4.00,5.00)			146(27.1%)/ (31.9%)	150(26.5%)/ (32.8%)	161(33.3%)/ (35.2%)		
How long did you spend on a cervical cancer screening? (*n* = 1586)								
<1 h	4.00(4.00,5.00)	21.841	**<0.001**	243(45.2%)/ (28.9%)	302(53.5%)/ (35.9%)	297(61.5%)/ (35.3%)	80.178	**<0.001**
1–2 h	5.00(4.00,5.00)			123(22.9%)/ (38.3%)	116(20.5%)/ (36.1%)	82(17.0%)/ (25.5%)		
3–4 h	4.00(4.00,5.00)			64(11.9%)/ (51.2%)	47(8.3%) (37.6%)	14(2.9%)/ (11.2%)		
>4 h	4.00(4.00,5.00)			108(20.1%) (36.2%)	100(17.7%)/ (33.6%)	90(18.6%)/ (30.2%)		
The level of awareness about HPV self-sampling and cervical cancer								
Low	4.00(3.00,5.00)	317.592	**<0.001**	282(44.0%)/ (45.4%)	203(31.5%)/ (32.7%)	136(26.8%)/ (21.9%)	45.593	**<0.001**
Medium	4.00(4.00,5.00)			230(35.9%)/ (31.7%)	257(39.9%)/ (35.4%)	239(47.0%)/ (32.9%)		
High	5.00(5.00,5.00)			129(20.1%)/ (28.9%)	184(28.6%)/ (41.3%)	133(26.2%)/ (29.8%)		
Experience of HPV self-sampling								
Low	3.00(3.00,3.00)	767.834	**<0.001**	105(16.4%)/ (56.5%)	39(6.1%)/ (21.0%)	42(8.3%)/ (22.6%)	88.201	**<0.001**
Medium	4.00(4.00,4.00)			320(49.9%)/ (41.8%)	239(37.1%)/ (31.2%)	207(40.7%)/ (27.0%)		
High	5.00(5.00,5.00)			216(33.7%)/ (25.7%)	366(56.8%)/ (43.5%)	259(51.0%)/ (30.8%)		

n(c)/(d): “n” means the number of people, “c” means the percentage of preference, and “d” means the percentage of the item

^a^ Kruskal—Wallis *H* test

^b^*χ2* test

### Results of multifactor regression analysis of HPV self-sampling awareness, experience, acceptability and preference

Multivariate regression analysis was performed with HPV self-sampling awareness, experience, acceptability and preference as dependent variables, and factors that served as independent variables were statistically significant in the univariate analysis. Tables 5, 6 and 7 present the results.

**Table 5:** Multiple linear regression analysis of the level of awareness and experience of HPV self-sampling

Variables		Awareness					Experience				
		B	SE	β	t	*P* value	B	SE	β	t	*P* value
Constants		23.217	0.620	-	37.451	**<0.001**	35.480	0.527	-	67.356	**<0.001**
Education	Junior High School	Ref	Ref	Ref	Ref	Ref	Ref	Ref	Ref	Ref	Ref
	Primary school or below	-0.957	0.762	-0.030	-1.256	0.209	0.376	0.663	0.014	0.567	0.571
	High school or vocational school	1.184	0.446	0.071	2.657	**0.008**	1.183	0.404	0.085	2.925	**0.003**
	Associate degree	1.676	0.439	0.110	3.817	**<0.001**	0.789	0.394	0.062	2.003	**0.045**
	Bachelor degree or above	1.876	0.496	0.122	3.782	**<0.001**	0.377	0.410	0.029	0.920	0.358
Marital status	Married						Ref	Ref	Ref	Ref	Ref
	Single						-0.757	1.227	-0.019	-0.617	0.537
	Divorced						0.508	0.707	0.017	0.719	0.472
	Widowed						-2.429	1.390	-0.041	-1.747	0.081
Fertility status	Have a child	Ref	Ref	Ref	Ref	Ref	Ref	Ref	Ref	Ref	Ref
	No child	0.269	0.798	0.008	0.337	0.736	-1.442	0.914	-0.049	-1.578	0.115
	Have 2 children or above	-0.256	0.307	-0.018	-0.835	0.404	-0.534	0.281	-0.046	-1.903	0.057
Abortion history	Never	Ref	Ref	Ref	Ref	Ref					
	1 abortion	-0.724	0.336	-0.052	-2.154	**0.031**					
	2 abortions or above	-0.519	0.376	-0.034	-1.381	0.167					
Monthly income per household	1000-3000RMB	Ref	Ref	Ref	Ref	Ref					
	<1000RMB	-2.183	0.927	-0.052	-2.355	**0.019**					
	3001-5000RMB	0.326	0.333	0.024	0.979	0.328					
	>5000RMB	0.359	0.429	0.021	0.837	0.403					
Occupations	Worker	Ref	Ref	Ref	Ref	Ref					
	Professional	2.012	0.571	0.091	3.521	**<0.001**					
	Service industry personnel	0.527	0.743	0.016	0.709	0.478					
	Company employee	0.671	0.358	0.047	1.871	0.061					
	Public functionary	1.351	0.758	0.039	1.782	0.075					
	Housewife	1.152	1.207	0.021	0.955	0.340					
	Retire	5.356	1.001	0.127	5.351	**<0.001**					
	Others	0.933	0.757	0.027	1.232	0.218					
Have you participated in the cervical cancer screening program conducted by the government or community?	Unheard	Ref	Ref	Ref	Ref	Ref	Ref	Ref	Ref	Ref	Ref
	Heard of it, not participated	1.725	0.403	0.121	4.276	**<0.001**	0.413	0.370	0.034	1.118	0.264
	Participated	2.405	0.433	0.179	5.547	**<0.001**	0.323	0.395	0.029	0.818	0.414
How recently did you get screened for cervical cancer?	<1 year	Ref	Ref	Ref	Ref	Ref	Ref	Ref	Ref	Ref	Ref
	1–2 years	-1.217	0.390	-0.070	-3.122	**0.002**	-0.054	0.354	-0.004	-0.153	0.878
	3–5 years	-3.151	0.829	-0.085	-3.802	**<0.001**	-1.233	0.756	-0.039	-1.631	0.103
	>5 years	-4.616	1.425	-0.072	-3.240	**0.001**	-2.234	1.283	-0.042	-1.742	0.082
	Never done it before	-5.156	0.598	-0.246	-8.623	**<0.001**	-2.021	0.546	-0.115	-3.705	**<0.001**
	Unclear	-4.256	1.118	-0.093	-3.806	**<0.001**	-1.816	1.020	-0.047	-1.780	0.075
What was the outcome of the most recent cervical cancer screening? (*n* = 1586)	Negative	Ref	Ref	Ref	Ref	Ref	Ref	Ref	Ref	Ref	Ref
	Positive	-0.649	0.686	-0.020	-0.946	0.344	-0.372	0.629	-0.014	-0.592	0.554
	Unclear	-2.400	0.736	-0.079	-3.262	**0.001**	-1.930	0.674	-0.076	-2.861	**0.004**
How much did you spend on a cervical cancer? (*n* = 1586)	For free	Ref	Ref	Ref	Ref	Ref	Ref	Ref	Ref	Ref	Ref
	<100RMB	-1.710	1.716	-0.022	-0.997	0.319	-2.124	1.571	-0.032	-1.352	0.177
	100-200RMB	-0.388	0.806	-0.011	-0.481	0.631	-1.740	0.735	-0.059	-2.366	**0.018**
	>200RMB	0.693	0.455	0.040	1.524	0.128	-0.684	0.415	-0.047	-1.649	0.099
	Unclear	-1.592	0.409	-0.104	-3.891	**<0.001**	-0.909	0.373	-0.070	-2.439	**0.015**
How long did you spend on a cervical cancer? (*n* = 1586)	<1 h	Ref	Ref	Ref	Ref	Ref	Ref	Ref	Ref	Ref	Ref
	1–2 h	0.163	0.408	0.009	0.400	0.689	0.151	0.372	0.010	0.407	0.684
	3–4 h	-1.780	0.612	-0.068	-2.908	**0.004**	0.153	0.561	0.007	0.273	0.785
	>4 h	-0.815	0.416	-0.045	-1.960	0.050	-0.209	0.380	-0.014	-0.549	0.583

**Table 6:** Multiple logistic regression analysis of HPV self-sampling acceptability

Acceptability	Variables		B	SE	Wald χ2	*P* value	OR	OR 95%CI
Low	The level of awareness about HPV self-sampling and cervical cancer		-0.083	0.023	12.512	**<0.001**	0.920	0.879–0.964
	Experience of HPV self-sampling		-0.313	0.031	99.543	**<0.001**	0.732	0.688–0.778
	Marital status	Married	Ref	Ref	Ref	Ref	Ref	Ref
		Single	-1.221	1.247	0.959	0.327	0.295	0.026–3.398
		Divorced	0.746	1.030	0.525	0.469	2.109	0.280-15.886
		Widowed	-1.498	0.807	3.446	0.063	0.224	0.046–1.087
	Fertility status	Have a child	Ref	Ref	Ref	Ref	Ref	Ref
		No child	1.138	1.228	0.859	0.354	3.120	0.281–34.638
		Have 2 children or above	-0.382	0.253	2.276	0.131	0.682	0.415–1.121
	Have you participated in the cervical cancer screening program conducted by the government or community?	Unheard	Ref	Ref	Ref	Ref	Ref	Ref
		Heard of it, not participated	0.717	0.310	5.361	**0.021**	2.048	1.116–3.756
		Participated	0.465	0.341	1.863	0.172	1.592	0.817–3.105
	How recently did you get screened for cervical cancer?	<1 year	Ref	Ref	Ref	Ref	Ref	Ref
		1–2 years	0.628	0.404	2.412	0.120	1.874	0.848–4.139
		3–5 years	-0.184	0.605	0.092	0.761	0.832	0.254–2.724
		>5 years	15.790	0.000	.	.	7203177.014	7203177.014
		Never done it before	-1.123	0.481	5.451	**0.020**	0.325	0.127–0.835
		Unclear	-0.091	0.696	0.017	0.896	0.913	0.233–3.571
	What was the outcome of the most recent cervical cancer screening? (*n* = 1586)	Negative	Ref	Ref	Ref	Ref	Ref	Ref
		Positive	-0.445	0.547	0.661	0.416	0.641	0.219–1.873
		Unclear	-1.085	0.482	5.062	**0.024**	0.338	0.131–0.870
	How much did you spend on a cervical cancer? (*n* = 1586)	For free	Ref	Ref	Ref	Ref	Ref	Ref
		<100RMB	-1.149	1.161	0.979	0.322	0.317	0.033–3.084
		100-200RMB	0.320	1.065	0.090	0.764	1.377	0.171–11.101
		>200RMB	-0.596	0.421	2.000	0.157	0.551	0.241–1.258
		Unclear	-0.977	0.362	7.276	**0.007**	0.376	0.185–0.766
	How long did you spend on a cervical cancer? (*n* = 1586)	<1 h	Ref	Ref	Ref	Ref	Ref	Ref
		1–2 h	0.129	0.379	0.116	0.733	1.138	0.542–2.390
		3–4 h	-0.306	0.472	0.419	0.518	0.737	0.292–1.859
		>4 h	-0.295	0.349	0.714	0.398	0.744	0.375–1.476
Medium	The level of awareness about HPV self-sampling and cervical cancer		-0.048	0.013	12.646	**<0.001**	0.953	0.928–0.979
	Experience of HPV self-sampling		-0.299	0.020	226.700	**<0.001**	0.742	0.713–0.771
	Marital status	Married	Ref	Ref	Ref	Ref	Ref	Ref
		Single	0.239	0.522	0.210	0.647	1.270	0.457–3.533
		Divorced	-0.232	0.328	0.499	0.480	0.793	0.417–1.509
		Widowed	-0.579	0.613	0.892	0.345	0.561	0.169–1.863
	Fertility status	Have a child	Ref	Ref	Ref	Ref	Ref	Ref
		No child	-0.699	0.380	3.375	0.066	0.497	0.236–1.048
		Have 2 children or above	-0.222	0.137	2.636	0.104	0.801	0.613–1.047
	Have you participated in the cervical cancer screening program conducted by the government or community?	Unheard	Ref	Ref	Ref	Ref	Ref	Ref
		Heard of it, not participated	-0.201	0.176	1.305	0.253	0.818	0.580–1.155
		Participated	0.021	0.199	0.011	0.917	1.021	0.692–1.507
	How recently did you get screened for cervical cancer?	<1 year	Ref	Ref	Ref	Ref	Ref	Ref
		1–2 years	-0.119	0.173	0.473	0.492	0.888	0.632–1.246
		3–5 years	0.154	0.390	0.155	0.693	1.166	0.543–2.504
		>5 years	15.451	0.000	.	.	5130099.277	5130099.277
		Never done it before	-0.623	0.240	6.716	**0.010**	0.536	0.335–0.859
		Unclear	-0.158	0.442	0.128	0.721	0.854	0.359–2.032
	What was the outcome of the most recent cervical cancer screening? (*n* = 1586)	Negative	Ref	Ref	Ref	Ref	Ref	Ref
		Positive	-0.282	0.302	0.872	0.350	0.754	0.417–1.363
		Unclear	-0.996	0.289	11.863	**0.001**	0.369	0.210–0.651
	How much did you spend on a cervical cancer? (*n* = 1586)	For free	Ref	Ref	Ref	Ref	Ref	Ref
		<100RMB	0.686	1.075	0.408	0.523	1.986	0.242–16.332
		100-200RMB	-0.265	0.347	0.581	0.446	0.767	0.388–1.516
		>200RMB	-0.038	0.209	0.034	0.854	0.962	0.639–1.448
		Unclear	-0.033	0.187	0.030	0.861	0.968	0.671–1.396
	How long did you spend on a cervical cancer? (*n* = 1586)	<1 h	Ref	Ref	Ref	Ref	Ref	Ref
		1–2 h	0.331	0.197	2.802	0.094	1.392	0.945–2.050
		3–4 h	0.001	0.274	0.000	0.997	1.001	0.585–1.711
		>4 h	-0.002	0.189	0.000	0.993	0.998	0.690–1.445

**Table 7:** Multiple logistic regression analysis of HPV self-sampling preferences

Preference	Variables		B	SE	Wald χ2	*p*-value	OR	OR 95%CI
Clinician-sampling	The level of awareness about HPV self-sampling and cervical cancer		-0.004	0.012	0.115	0.735	0.996	0.972–1.020
	Experience of HPV self-sampling		-0.067	0.014	23.771	**<0.001**	0.935	0.911–0.961
	Age	≤ 34 years old	Ref	Ref	Ref	Ref	Ref	Ref
		35–44	0.074	0.183	0.163	0.687	1.077	0.752–1.541
		45–54	0.567	0.211	7.230	**0.007**	1.762	1.166–2.663
		≥ 55	0.302	0.566	0.285	0.593	1.353	0.446–4.098
	Education	Junior High School	Ref	Ref	Ref	Ref	Ref	Ref
		Primary school or below	-2.534	1.041	5.924	**0.015**	0.079	0.010–0.611
		High school or vocational school	0.835	0.213	15.316	**<0.001**	2.305	1.517–3.503
		Associate degree	0.889	0.223	15.831	**<0.001**	2.432	1.570–3.768
		Bachelor degree or above	1.181	0.243	23.643	**<0.001**	3.258	2.024–5.244
	Fertility status	Have a child	Ref	Ref	Ref	Ref	Ref	Ref
		No child	0.572	0.362	2.491	0.115	1.772	0.871–3.605
		Have 2 children or above	-0.131	0.148	0.784	0.376	0.877	0.656–1.172
	Abortion history	Never	Ref	Ref	Ref	Ref	Ref	Ref
		1 abortion	-0.495	0.158	9.844	**0.002**	0.610	0.447–0.830
		2 abortions or above	-0.276	0.175	2.468	0.116	0.759	0.538–1.071
	Monthly income per household	1000-3000RMB	Ref	Ref	Ref	Ref	Ref	Ref
		<1000RMB	-0.624	0.480	1.691	0.194	0.536	0.209–1.372
		3001-5000RMB	-0.378	0.151	6.308	**0.012**	0.685	0.510–0.920
		>5000RMB	-0.270	0.205	1.729	0.188	0.763	0.510–1.142
	Occupations	Worker	Ref	Ref	Ref	Ref	Ref	Ref
		Professional	-0.979	0.252	15.052	**<0.001**	0.376	0.229–0.616
		Service industry personnel	0.180	0.316	0.325	0.568	1.198	0.645–2.225
		Company employee	-0.487	0.168	8.385	**0.004**	0.615	0.442–0.854
		Public functionary	0.338	0.339	0.996	0.318	1.403	0.722–2.726
		Housewife	-1.239	0.830	2.229	0.135	0.290	0.057–1.473
		Retire	-0.179	0.694	0.066	0.797	0.836	0.215–3.257
		Others	-0.467	0.370	1.586	0.208	0.627	0.303–1.296
	Have you participated in the cervical cancer screening program conducted by the government or community?	Unheard	Ref	Ref	Ref	Ref	Ref	Ref
		Heard of it, not participated	0.283	0.194	2.136	0.144	1.327	0.908–1.940
		Participated	0.104	0.207	0.250	0.617	1.109	0.739–1.665
	How recently did you get screened for cervical cancer?	<1 year	Ref	Ref	Ref	Ref	Ref	Ref
		1–2 years	-0.380	0.181	4.378	**0.036**	0.684	0.479–0.976
		3–5 years	-0.229	0.427	0.287	0.592	0.795	0.344–1.838
		>5 years	-0.679	0.842	0.651	0.420	0.507	0.097–2.640
		Never done it before	-1.352	0.309	19.189	**<0.001**	0.259	0.141–0.474
		Unclear	-1.093	0.604	3.277	0.070	0.335	0.103–1.095
	What was the outcome of the most recent cervical cancer screening? (*n* = 1586)	Negative	Ref	Ref	Ref	Ref	Ref	Ref
		Positive	-0.520	0.290	3.207	0.073	0.595	0.337–1.050
		Unclear	0.210	0.346	0.367	0.545	1.233	0.626–2.431
	How much did you spend on a cervical cancer? (*n* = 1586)	For free	Ref	Ref	Ref	Ref	Ref	Ref
		<100RMB	-1.169	1.144	1.044	0.307	0.311	0.033–2.925
		100-200RMB	-2.131	0.562	14.381	**<0.001**	0.119	0.039–0.357
		>200RMB	-0.684	0.212	10.415	**0.001**	0.505	0.333–0.765
		Unclear	0.017	0.181	0.008	0.927	1.017	0.712–1.451
	How long did you spend on a cervical cancer? (*n* = 1586)	<1 h	Ref	Ref	Ref	Ref	Ref	Ref
		1–2 h	-0.281	0.184	2.323	0.127	0.755	0.526–1.084
		3–4 h	-1.014	0.334	9.191	**0.002**	0.363	0.188–0.699
		>4 h	-0.272	0.183	2.196	0.138	0.762	0.532–1.092
Self-sampling	The level of awareness about HPV self-sampling and cervical cancer		-0.013	0.012	1.222	0.269	0.987	0.964–1.010
	Experience of HPV self-sampling		0.031	0.013	5.515	**0.019**	1.031	1.005–1.058
	Age	≤ 34 years old	Ref	Ref	Ref	Ref	Ref	Ref
		35–44	0.091	0.171	0.284	0.594	1.095	0.784–1.531
		45–54	0.601	0.200	9.047	**0.003**	1.823	1.233–2.697
		≥ 55	-0.094	0.542	0.030	0.862	0.910	0.315–2.632
	Education	Junior High School	Ref	Ref	Ref	Ref	Ref	Ref
		Primary school or below	-2.690	1.040	6.691	**0.010**	0.068	0.009–0.521
		High school or vocational school	0.311	0.214	2.105	0.147	1.364	0.897–2.076
		Associate degree	0.206	0.222	0.865	0.352	1.229	0.796–1.898
		Bachelor degree or above	0.509	0.239	4.549	**0.033**	1.664	1.042–2.657
	Fertility status	Have a child	Ref	Ref	Ref	Ref	Ref	Ref
		No child	0.480	0.345	1.935	0.164	1.617	0.822–3.181
		Have 2 children or above	-0.030	0.142	0.044	0.834	0.971	0.735–1.282
	Abortion history	Never	Ref	Ref	Ref	Ref	Ref	Ref
		1 abortion	-0.069	0.150	0.213	0.644	0.933	0.696–1.251
		2 abortions or above	0.108	0.168	0.410	0.522	1.114	0.801–1.550
	Monthly income per household	1000-3000RMB	Ref	Ref	Ref	Ref	Ref	Ref
		<1000RMB	-0.261	0.518	0.254	0.614	0.770	0.279–2.128
		3001-5000RMB	-0.315	0.146	4.658	**0.031**	0.730	0.548–0.971
		>5000RMB	-0.657	0.191	11.775	**0.001**	0.519	0.356–0.755
	Occupations	Worker	Ref	Ref	Ref	Ref	Ref	Ref
		Professional	-0.082	0.256	0.103	0.748	0.921	0.557–1.522
		Service industry personnel	0.607	0.332	3.355	0.067	1.835	0.958–3.516
		Company employee	-0.286	0.161	3.175	0.075	0.751	0.548–1.029
		Public functionary	0.329	0.302	1.182	0.277	1.389	0.768–2.513
		Housewife	-1.462	0.798	3.352	0.067	0.232	0.048–1.109
		Retire	-1.155	0.630	3.356	0.067	0.315	0.092–1.084
		Others	-0.282	0.352	0.642	0.423	0.754	0.378–1.504
	Have you participated in the cervical cancer screening program conducted by the government or community?	Unheard	Ref	Ref	Ref	Ref	Ref	Ref
		Heard of it, not participated	0.326	0.191	2.914	0.088	1.385	0.953–2.014
		Participated	0.042	0.202	0.043	0.835	1.043	0.702–1.548
	How recently did you get screened for cervical cancer?	<1 year	Ref	Ref	Ref	Ref	Ref	Ref
		1–2 years	-0.379	0.176	4.601	**0.032**	0.685	0.485–0.968
		3–5 years	-0.319	0.429	0.553	0.457	0.727	0.313–1.685
		>5 years	-0.379	0.872	0.189	0.664	0.684	0.124–3.778
		Never done it before	-0.983	0.306	10.340	**0.001**	0.374	0.205–0.681
		Unclear	-0.546	0.639	0.730	0.393	0.579	0.165–2.027
	What was the outcome of the most recent cervical cancer screening? (*n* = 1586)	Negative	Ref	Ref	Ref	Ref	Ref	Ref
		Positive	0.204	0.320	0.407	0.523	1.226	0.655–2.295
		Unclear	0.162	0.350	0.215	0.643	1.176	0.592–2.337
	How much did you spend on a cervical cancer? (*n* = 1586)	For free	Ref	Ref	Ref	Ref	Ref	Ref
		<100RMB	-0.918	1.169	0.617	0.432	0.399	0.040–3.949
		100-200RMB	-1.456	0.575	6.403	**0.011**	0.233	0.076–0.720
		>200RMB	-0.327	0.208	2.476	0.116	0.721	0.480–1.084
		Unclear	0.128	0.172	0.551	0.458	1.136	0.811–1.592
	How long did you spend on a cervical cancer? (*n* = 1586)	<1 h	Ref	Ref	Ref	Ref	Ref	Ref
		1–2 h	-0.102	0.179	0.323	0.570	0.903	0.636–1.283
		3–4 h	-0.780	0.338	5.326	**0.021**	0.458	0.236–0.889
		>4 h	-0.031	0.178	0.030	0.863	0.970	0.684–1.375

The level of awareness was greater for high school and above (*B* = 1.184, *P* = 0.008; *B* = 1.676, *P* < 0.001; *B* = 1.876, *P* < 0.001), professionals (*B* = 2.012, *P* = 0.031), retired people (*B* = 5.356, *P* < 0.001), and people with previous screening experience (*B* = 2.405, *P* < 0.001), while low income (*B*=-2.183, *P* = 0.019) was associated with a lower level of awareness. Individuals with a high school education (*B* = 1.183, *P* = 0.003) and associate education (*B* = 0.789, *P* = 0.045) had better experience. Those who had never been screened (*B*=-5.156, *P* < 0.001), as well as those who were unfamiliar with previous cervical cancer screening (*B* < 0, *P* < 0.001), had poorer experience. The level of awareness, experience, inadequate cervical cancer screening, and unfamiliarity with previous cervical cancer screening all affected acceptability (*P* < 0.05).A greater level of HPV self-sampling experience was related to greater self-sampling preference (*B* = 0.031, *P* = 0.019). Those aged 45–54 years demonstrated a preference for both choices, and they chose clinician-sampling (*OR* = 1.762 (1.116–2.163)) or self-sampling (*OR* = 1.823 (1.233–2.697)) 1.8 times younger (age 34 and younger). Those with a high school education and above (*OR* = 2.305 (1.517–3.503), *OR* = 2.432 (1.570–3.768), *OR* = 3.258 (2.024–5.244))chose clinician sampling 2.3–3.3 times more than did those with a junior high school education, and those with a bachelor’s degree or above (*OR* = 1.664 (1.042–2.657)) chose self-sampling 1.7 times more than did those with a junior high school education. Middle- and high-income groups did not show a preference for either of these sampling methods. The middle-income group chose clinician sampling (*OR* = 0.685 (0.510–0.920)) or self-sampling (*OR* = 0.730 (0.548–0.971)) 0.7 times that of the low-income group, and the high-income group chose self-sampling (*OR* = 0.519(0.356–0.755)) 0.5 times that of the low-income group.

## Discussion

### Awareness

The mean total score for the level of awareness in this study was 24.6 out of 35, which is moderate. Haward et al. surveyed Canadian women and discovered that their understanding of HPV self-sampling was inadequate, with a correct knowledge rate of only 20.4% [24]. In contrast, a cross-sectional survey in Brazil showed that 70% of the participants correctly answered four-fifths of the HPV knowledge questions [25], which is a high level of knowledge. It is possible that there were large differences in the level of awareness due to the heterogeneity of the questionnaires used to assess knowledge across studies. In our study, understanding of the HPV self-sampling process received the best score among all the questions of knowledge, which may be related to the fact that the participants were women who had engaged in self-sampling. Knowledge of the methods for addressing HPV-positivity received the lowest grade, and at the same time, many participants also gave feedback that they hoped the positive report could be interpreted and the next step in dealing with this could be given, which echoed the current problems of insufficient processing of cervical cancer after the initial screening. This finding suggests that we need to strengthen the triage management of people with positive initial screening results for cervical cancer, improve the systematic standardization process, and ensure the effective tracking and follow-up of these people.

This study revealed that a high school degree or above and professional or retired status positively influence the level of awareness, and the greater the education level is, the greater the positive influence of the relationship. This finding is in line with the majority of the study findings. According to some studies, social class and years of education are related to one’s comprehension of cervical cancer screening [25]. Professionals (e.g., accountants, lawyers, architects, healthcare professionals, journalists, etc.) generally have a higher socioeconomic status and higher level of education, and individuals with more educational attainment may be more willing to acquire and learn about the relevant knowledge and understand the necessity of preventive healthcare better. In addition, retired people are well experienced and may have a better understanding of cervical cancer screening and self-sampling for HPV. Regarding income, a family’s monthly earnings per capita of no more than 1,000 RMB had a negative impact on the level of awareness, probably because low-income people neglect to pursue a good quality of life and do not pay enough attention to their own health and cervical cancer screening. Education is closely linked to health habits, and academic performance influences health by determining chances, job opportunities, and earnings, providing individuals with better economic empowerment and thus access to high-quality health care [26]. This study revealed that a history of cervical cancer screening was linked to the level of awareness. Hearing about a cervical cancer screening programme organized by the government and community had a positive impact on the level of awareness, and participants had a greater positive impact than nonparticipants. In contrast, never having been screened, having undergone the most recent screening more than a year ago, being unfamiliar with previous cervical cancer screening, had a negative impact on the level of awareness, and longer intervals between the most recent screenings had greater negative impacts. This finding suggested that individuals who had in-person screening experience had a greater level of awareness, which may be related to the information gained about HPV during testing. Previous studies have reported similar results. A national survey in the United States reported that knowing about HPV was linked to having had a test for it within the past 5 years [14]. Cervical cancer, HPV, and HPV test knowledge were assessed in a Canadian study, which showed that there was a noticeable decrease in knowledge among those who were underscreened compared to those who were adequately screened [24]. With changes in clinical recommendations and scientific developments, more scientifically based questionnaires with more comprehensive reliability tests need to be developed to measure HPV self-sampling knowledge someday to explore the effect that knowledge has on anticipating cervical cancer screening activities.

### Experience

The mean total score for experience in this study was 35.4 out of 45, which is moderate. Among all the questions about HPV self-sampling experience, privacy level was the highest, and pain level was the lowest. These findings are consistent with previous studies reporting mild pain and less embarrassment with self-sampling [27, 28]. Our study showed that literacy was associated with experience. A high school education and associate degree had a positive effect on experience, but a bachelor’s degree and above had no effect. It is possible that women with an upper middle level of education are better able to understand and master the self-sampling procedure, which in turn leads to a better experience. Women with a bachelor’s degree or above may have greater expectations of self-sampling experience due to their higher level of education. Neither having been screened nor being unfamiliar with previous cervical cancer screening (including being unclear about the results of the most recent screening and the cost of having a screening) had a negative impact on experience. This may be because people have no previous experience with screening or have rejected screening, do not take screening seriously enough, and subsequently have poor experience. HPV self-sampling experience is related to women’s participation and motivation for this screening approach, which may affect the feasibility of its practical application. In the future, the screening experience needs to be refined to include all steps of the self-sampling process to optimize the screening technique.

### Acceptability and preference

The level of acceptability in this study was high, with 88.8% of participants finding self-sampling acceptable. In terms of preference, the majority (76.9%) of participants were ready to introduce HPV self-sampling to others, which is consistent with the findings of Parker et al., who reported that 73.8% of self-sampling kit users in the United States would recommend it to friends [29]. In our study, self-sampling was preferred by 64.2% of participants for cervical cancer screening, which is slightly lower than that reported in other studies. A survey conducted in Jiangsu Province, China, showed that 73% of women were ready to choose self-samples [30]. A study in Hong Kong, China, reported a preference of 69% for self-sampling [31], and 65% for self-sampling in Yunnan, China [32]. A global systematic evaluation reported a range of self-sampling choices from 50–93% [8]. Many previous studies have categorized self-sampling preference as acceptable, but our study described acceptability separately from preference; thus, there was a high level of acceptability but a low level of preference, which may be related to the inclusion of clinician sampling as an option. Aitken et al. noted that fewer women chose self-sampling when there was clinician sampling [33]. The main reasons for choosing clinician-sampling in our study included more accurate sampling, more disease information visible to the clinician, and more reliable results. The main reasons for choosing self-sampling included convenience, easy operation, better privacy, and less fear and pain associated with the gynecological speculum. This finding is similar to the results of previous studies [8, 24]. In addition, the level of awareness, experience, lack of cervical cancer screening (including never having been screened, hearing about cervical cancer screening programs but not attending a screening), and unfamiliarity with previous cervical cancer screenings (including being unclear about the results of the most recent screening and the cost of a screening) all affect acceptability.

As far as the influencing factors on preference are concerned, the experience of HPV self-sampling has an effect on both choosing clinician sampling and self-sampling. The more experience one has, the more ready one is to select self-sampling. However, the level of awareness has no effect on preferences. The effect of practice on attitudes may be somewhat greater than the effect of knowledge on attitudes. Further studies revealed that never having been screened and completing a screening session were time-consuming (in the range of 3 to 4 h) and had a negative impact on both choices, possibly due to the participants’ own low acceptability of cervical cancer screening and poor previous screening experience. This led us to identify differences from the findings of other studies, where women who had not been screened were more inclined to self-samples [31, 34].

#### Age

Age is a consideration for cancer screening beliefs or adherence to a screening program. In our study, participants aged 45 to 54 years showed a preference for both options, choosing either clinician sampling or self-sampling 1.8 times more often than younger participants did(aged 34 and younger), which may be related to a stronger willingness to screen in older women. Previous studies on the effect of age have yielded mixed results. Drysdale et al. surveyed women in the UK and reported that their preference for self-sampling increased with age [35]. A US study on cancer screening methods also revealed lower screening rates for cervical cancer in younger age groups [36]. In contrast, a survey of underscreened women in Hong Kong, China, showed that young women aged 25 to 35 years were substantially more likely to prefer self-sampling than women aged ≥ 45 years [31].

#### Education

In terms of literacy, those with a high school education or above chose clinician sampling 2.3 to 3.3 times more often than did those with a junior high school education, and those with a bachelor’s degree or above chose self-sampling 1.7 times more often than did those with a junior high school education, whereas primary school education or below negatively influenced both choices. It is possible that people with an upper middle level of education are better able to understand the significance of the choice of screening methods. More highly educated individuals, however, could be more inclined to select self-sampling than clinician sampling. This differs from the findings of many current studies. One study revealed that highly educated women were less confident in self-sampling and questioned their ability to self-samples, thus preferring clinician sampling [37, 38]. However, a different investigation of vaginal HPV self-sampling revealed no correlation between self-sampling preference and educational background [34].

#### Income

It has been demonstrated that income or economic level may influence cervical cancer screening rates and modality selection, with high-income nations having significantly higher cervical cancer screening rates than low- and middle-income nations [39]. However, our study drew interesting conclusions. Middle- and high-income groups did not show a preference for either of these sampling methods. For middle-income individuals, clinician sampling or self-sampling was chosen at 0.7 times the rate for low-income individuals, and for high-income individuals, self-sampling was chosen at 0.5 times the rate for low-income individuals. Given that both sampling methods have their own drawbacks and may not meet the expectations of middle- and upper-income populations, future optimization and innovation in screening techniques are needed.

#### Concerns about HPV self-sampling

The primary concern participants in our study had about HPV self-sampling was deterioration or contamination, which may be related to the fact that the specimens after completing self-sampling were not tested directly but rather needed to be sent for testing by the participants themselves via postal couriers. Some participants expressed a desire to perform self-sampling in the hospital, to test as they were collected, and to ensure the quality of the specimen. Most related studies have been performed with courier-based self-sampling kits [40], and fewer studies have evaluated hospital-based HPV self-sampling [41]. Hospital self-sampling may help to address problems associated with lost specimens, sample contamination and other issues with postal specimens, as well as inquiries on self-sampling methods (e.g., guidance given on-site via hospital professionals). However, our survey of self-sampling places showed that more than half (56.4%) of the participants said they would rather self-sample at home, and 39.5% preferred self-sampling in a hospital. This may be related to the fact that home is more comfortable and provides more privacy, whereas going to the hospital is more cumbersome and increases the cost of time and money. It is possible that participants would give more consideration to their own sense of experience and cost-effectiveness compared to the quality of the specimen. Other studies have shown similar preferences for home sampling. In a Belgian study assessing attitudes and experiences with self-sampling, self-sampling at home was preferred by 57% of participants over collection by a physician [42]. A randomized three-arm experiment in New Zealand noted a significantly greater participation rate for HPV self-sampling for testing at home than for routine sampling in a clinic [43]. The second major concern is incorrect sampling. Although this study provided targeted education and counseling services via video/illustration/text at each step of the cervical HPV self-sampling process, enabling participants to access sample kits and sampling instructions through the platform, participants continued to lack confidence in the implementation of self-sampling, and reported concerns about lacking interpretation of the results and recommendations for treatment. The third major concern was inaccurate results. Although many studies have indicated that self-sampling and clinician-sampling yield comparable test results, women still have great concerns regarding the correctness of self-sampling results. This suggests that accurate and comprehensive information is critical to the successful implementation of self-sampling. Women’s opinions are influenced by the experience of the test, as well as by the justification for self-sampling (e.g., awareness of the connection between HPV and cervical cancer, and the significance of a positive outcome) [44]. A qualitative investigation into the knowledge and attitudes of Pacific Islander women regarding cervical cancer screening revealed the necessity for information that is easily accessible, sufficiently accurate, and consistent to increase women’s self-sampling belief and guarantee that they receive their test results in a format that makes sense to them [45]. This suggests that we should educate individuals before carrying out self-sampling tests, emphasizing the similar fidelity of self-sampling to that of the clinician sample, and answer the participants’ questions, and ensure that the instructions in the self-sampling process are clear and easy to understand [5]. Based on these findings, other related studies have reported similar concerns [5, 7]. In addition, participants in our study were concerned about not receiving results in a timely manner, with the majority (92.5%) of them wanting to report on the HPV self-sampling test within one week. This finding suggested that we need to improve the testing process and technology to speed up testing. A total of 67.7% of the participants were willing to pay no more than 100 RMB for the self-sampling test, and some of them gave feedback that they would like to see the test reduced in price or even free of charge. A Nepalese study also showed that women were more inclined to undergo self-sampling tests when self-sampling kits were more reasonably priced [46]. Based on the cost-effectiveness of self-sampling tests, simplification of screening programs is a prerequisite for successful self-sampling. In turn, the target population’s sociodemographic status, the interval between tests, the preselected HPV molecular platform, and the triage approach are the primary factors influencing cost-effectiveness [47].

In the last open-ended question in the questionnaire of our study, participants provided more feedback on the following aspects: (1) the accuracy of the results of self-sampling; (2) the timeliness of the specimen, hoping to accelerate the speed of testing; (3) the comprehensiveness and readability of the test report, hoping to interpret the positive report and give the next step in the treatment; (4) suggestions to increase the frequency of the test, 1 to 2 times a year; (5) suggestions to popularize cervical cancer and HPV screening knowledge; and (6) the accessibility of the test, hoping to reduce the price or free screening. In response to feedback from many participants in our study that they would like to increase the frequency of testing, a similar willingness to screen was reported in a Brazilian study, where 93.8% of the participants felt that cytology should be performed more frequently than recommended [25]. However, it has been demonstrated that HPV testing is more sensitive than cytology or visual inspection based on acetic acid, allowing longer screening intervals. The latest WHO guidelines recommend regular screening every 5 to 10 years among women in general when using HPV DNA testing as the primary screening method [3]. This finding suggested that education should be strengthened, women’s perceptions of the frequency of testing should be improved, and medical resources should be avoided due to overscreening.

### Limitations

There are several limitations to our research. First, given that this study was cross-sectional, the use of self-reported screening may be affected by memory bias, limiting the applicability of the findings. For the study subjects, convenience samples never result in a statistically balanced selection of the population; 66.5% of them were from Jiangsu, which was not equally distributed among the three regions, leading to selection bias. Second, in terms of questionnaire design, sociodemographic characteristics were not related to ethnicity (although China is an ethnically diverse country with 56 ethnic groups); sexual orientation (based on the traditional Chinese concept of prevalence of heterosexuality, we did not include homosexuality, bisexuality, etc.). The assessment of knowledge was not comprehensive, e.g., awareness of cervical cancer symptoms or the connection between HPV and the disease were not addressed. Third, the HPV self-sampling method used in this study was limited to vaginal samples and did not include urine samples. Finally, there was no follow-up management of women who tested positive for HPV via self-sampling. Despite these drawbacks, our research still evaluated in detail the relationships between sociodemographic variables, past cervical cancer screening history, and attitudes and knowledge regarding HPV self-sampling.

## Conclusions

In this study, the level of awareness and experience of HPV self-sampling among women were moderate, with high acceptability but low preference, indicating that HPV self-sampling is feasible and that related knowledge and experience need to be improved. In the actual use of self-sampling, it is necessary to continually compare self-sampling with traditional clinician sampling to expand the strengths and compensate for the deficiencies. The main obstacles to promoting HPV self-sampling and increasing preferences should be addressed by optimizing screening technology and overcoming differences in knowledge and screening perceptions. Increasing public awareness of HPV testing and cervical cancer screening should be integrated into daily life to subconsciously influence people’s perceptions. It is important to achieve full coverage of those eligible for screening while avoiding frequent overscreening during the screening process and ensuring follow-up treatment of individuals whose screening findings are abnormal.

The results of this study may help to adjust public health strategies for the early inclusion of HPV self-sampling as a screening method in national initiatives to prevent cervical cancer. In the future, additional research will be needed to extend the findings to other countries and other regions of China, to address the challenges of never- and underscreening and to help achieve the WHO’s goal of completely eradicating cervical cancer as a global public health issue by 2030.

## Data Availability

The raw data of the participants are protected and are not available due to data privacy laws. The processed data are, however, available from the author Jian-Fen Qin(3193160@zju.edu.cn) upon reasonable request and with the permission of Ethics Committee of Sir Run Run Shaw Hospital, Zhejiang University School of Medicine.

## Abbreviations

BMI: Body mass index
HPV: Human Papillomavirus
IARC: The International Agency for Research on Cancer
PCR: Polymerase chain reaction
WHO: The World Health Organization
